# ESTIMATING AGE-SPECIFIC HAZARD RATES OF INFECTION FROM CROSS-SECTIONAL OBSERVATIONS

**DOI:** 10.15517/rmta.v27i1.39952

**Published:** 2019-12-17

**Authors:** ZHILAN FENG, JOHN W. GLASSER

**Affiliations:** * Purdue University, Department of Mathematics, West Lafayette IN, United States.; † National Center for Immunization and Respiratory Diseases, CDC, Atlanta GA, United States.

**Keywords:** epidemiological model, force of infection, parameter estimation, cross-sectional observations, serology data, modelo epidemiológico, fuerza de infección, estimación de parámetros, observaciones transversales, datos serológicos, 34C99, 35Q92, 92B05

## Abstract

Mathematical models of pathogen transmission in age-structured host populations, can be used to design or evaluate vaccination programs. For reliable results, their forces or hazard rates of infection (FOI) must be formulated correctly and the requisite contact rates and probabilities of infection on contact estimated from suitable observations. Elsewhere, we have described methods for calculating the probabilities of infection on contact from the contact rates and FOI. Here, we present methods for estimating the FOI from cross-sectional serological surveys or disease surveillance in populations with or without concurrent vaccination. We consider both continuous and discrete age, and present estimates of the FOI for vaccine-preventable diseases that confer temporary or permanent immunity.

## Resumen

Los modelos matemáticos de transmisión de patógenos en poblaciones de huéspedes estructuradas por edad pueden usarse para diseñar o evaluar programas de vacunación. Para obtener resultados confiables, sus fuerzas o tasas de riesgo de infección (FOI) deben formularse correctamente y las tasas de contacto requeridas y las probabilidades de infección en contacto deben estimarse a partir de observaciones adecuadas. En otros lugares, hemos descrito métodos para calcular las probabilidades de infección por contacto a partir de las tasas de contacto y FOI. Aquí, presentamos métodos para estimar el FOI a partir de encuestas serológicas transversales o vigilancia de enfermedades en poblaciones con o sin vacunación concurrente. Consideramos tanto la edad continua como la discreta, y presentamos estimaciones del FOI para enfermedades prevenibles por vacunación que confieren inmunidad temporal o permanente.

## 1 Introduction

Vaccine-preventable diseases such as measles and pertussis have age-specific vaccination programs. Epidemiological models can be used to identify target age groups (e.g., Hao et al. 2019, [6]). For such models to generate reliable evaluations of alternative strategies, they must have reasonable parameter estimates. Among model parameters, the most important and difficult to estimate is the probability of infection per contact, usually denoted by *β*, which is the main component of the FOI.

Consider an age-structured SIR model with continuous age and assume that the system is at the endemic steady-state. Let *S*(*α*) denote the density of susceptible people aged *α*. Denote the number of new infections by *λ*(*α*)*S*(*α*), where *λ*(*α*) is the FOI given by

(1) $$\lambda (\alpha )=a(\alpha )\beta (\alpha )\int_{0}^{\infty }c(\alpha ,u)\frac{I(u)}{N(u)}du.$$

In equation (1), *a*(*α*) denotes the *per capita* contact rate of individuals aged *α*, *β*(*α*) is the probability of infection per contact among susceptible ones aged *α*, *c*(*α*, *u*) describes mixing between susceptible and infectious people aged *α* and *u*, respectively, and *I*(*u*) and *N*(*u*) denote the densities of infectious individuals and total population aged *u*, respectively. Their ratio is the probability that a randomly encountered person aged *u* is infectious.

If the population can be divided into *n* age groups such that the characteristics of individuals within each are the same, then Hethcote (2000) showed that models comprising partial differential equations (PDEs) can be reduced to systems of ordinary differential equations (ODEs) with *n* discrete age groups indexed by *i* = 1, 2, …, *n*. In this case, the number of new infections in age group *i* is *λ*_*i*_*S*_*i*_, where *S*_*i*_ denotes the number of susceptible individuals in group *i* and *λ*_*i*_ is the FOI for that group (Section 2.2):

(2) $$\lambda_{i}=a_{i}\beta_{i}\overset{n}{\underset{j=1}{\sum }}c_{ij}\frac{I_{j}}{N_{j}},\;i=1,2,\ldots ,n.$$

The parameter values for the contact rates *a*_*i*_ and proportions *c*_*ij*_ can be estimated from observed contacts between age-groups (see [5, 2]). Using estimates of *λ*_*i*_ and *I*_*j*_/*N*_*j*_, we can solve the equations in (2) for the probabilities of transmission *β*_*i*_.

## 2 Linking the FOI to observations

Serological observations may include individuals with immunity induced by vaccination as well as natural infection. As these sources generally are indistinguishable, additional information about vaccination programs is needed to estimate the FOI from post-vaccination serological observations.

### 2.1 Continuous age

Let *α* denote chronological age, *F*(*α*) denote the cumulative probability of being infected at age *α*, and *λ*(*α*) denote the *per capita* infection rate for susceptible individuals aged *α*. The probability that a person remains susceptible from birth to age *α* is $e^{-\int_{0}^{\alpha }\lambda (s)ds}$, so

(3) $$F(\alpha )=1-e^{-\int_{0}^{\alpha }\lambda (s)ds}.$$

**Figure 1:**
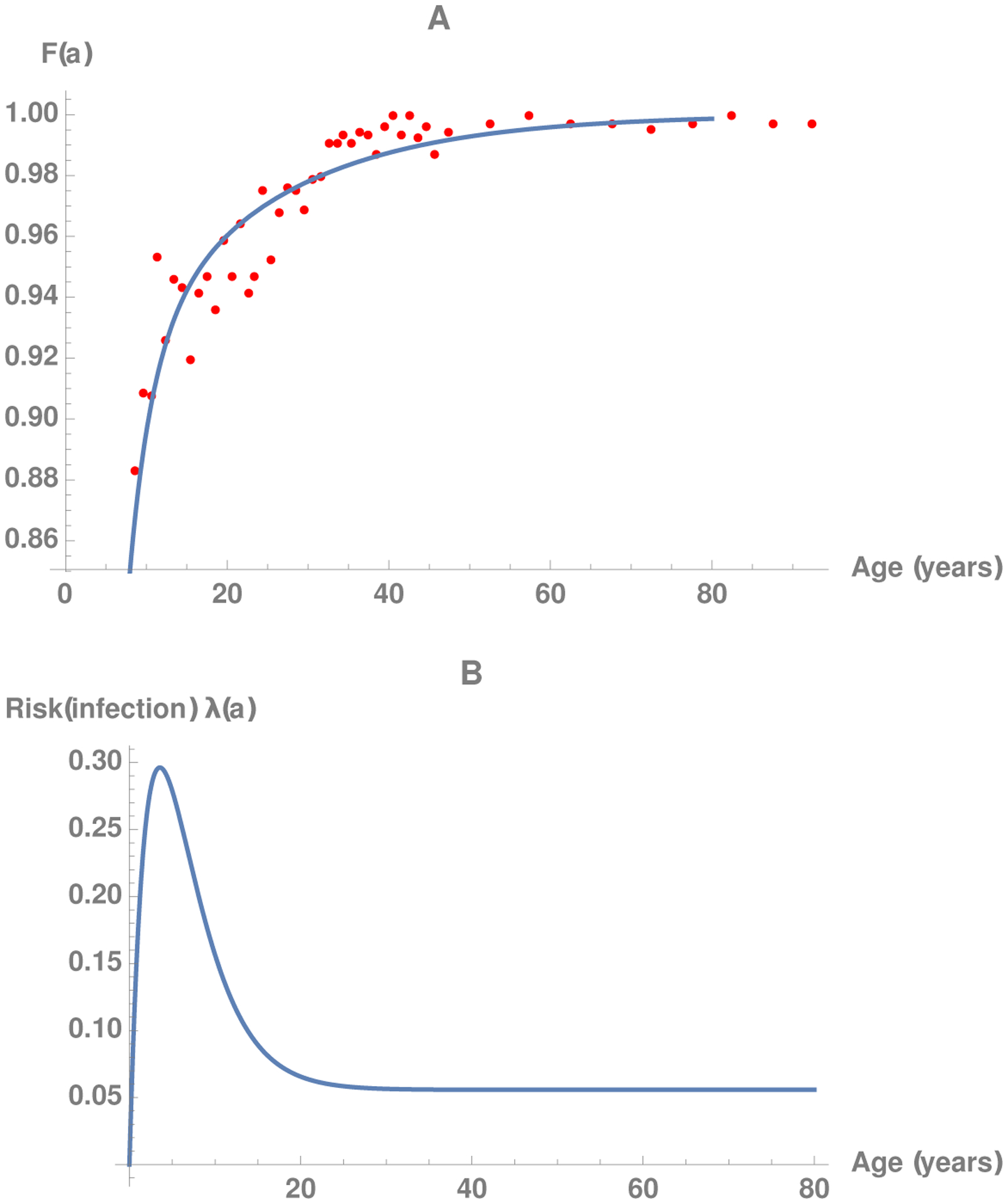
A: Serological observations (dotted) for varicella (from NHANES III, conducted from 1988–1995) and the fitted curve (solid) for *F*(*α*). B: The FOI *λ*(*a*) calculated from the fitted *F*(*α*) in A and (4).

In the absence of vaccination, *F*(*α*) can be obtained directly from serological observations. For example, Figure 1A illustrates *F*(*α*) fitted to observations (represented by the dots) for varicella from the third National Health and Nutrition Examination Survey (https://www.cdc.gov/nchs/nhanes/nh3data.htm), conducted in the United States during the period 1988–1995, via the FindFit function in Mathematica. A vaccine against varicella was not licensed in the US until 1995. Using the estimated function *F*(*α*) and relation (3), we can obtain the FOI as follows:

(4) $$\lambda (\alpha )=-\frac{d}{d\alpha }\;\text{ln}\;[1-F(\alpha )].$$

A plot of *λ*(*α*) is illustrated in Figure 1B.

If vaccination at birth (or soon after) is considered, let *q*(*α*) denote the fraction of individuals aged *α* who were *not* immunized at birth. Then the expression for *F* in (3) becomes

(5) $$F(\alpha )=1-q(\alpha )e^{-\int_{0}^{\alpha }\lambda (s)ds},$$

and the corresponding FOI is given by

(6) $$\lambda (\alpha )=\frac{d}{d\alpha }\;\text{ln}\;\frac{q(\alpha )}{1-F(\alpha )}.$$

### 2.2 Discrete age

If the population can be divided into *n* subgroups by 0 = *α*_0_ < *α*_1_< ⋯ <*α*_*n*_ = ∞, such that parameter values within each group [*a*_*i*−1_, *a*_*i*_) are constant; that is,

$$a(\alpha )=a_{i},\beta (\alpha )=\beta_{i},\;\text{etc.}\;\alpha_{i-1}\leq \alpha <\alpha_{i},i=1,2,\ldots ,n,$$

the numbers of individuals in the respective epidemiological classes in age group *i*, *α*_*i*−1_ ≤ *α* < *α*_*i*_, are

$$S_{i}=\int_{\alpha_{i-1}}^{\alpha_{i}}S(\alpha )d\alpha ,\;I_{i}=\int_{\alpha_{i-1}}^{\alpha_{i}}I(\alpha )d\alpha ,\;N_{i}=\int_{\alpha_{i-1}}^{\alpha_{i}}N(\alpha )d\alpha ,\;i=1,2,\ldots ,n.$$

If the mixing function *c*(*α*, *u*) is separable and properly defined (see [4]), it can be replaced by discrete mixing constants *c*_*ij*_, representing the proportion of the contacts of individuals in group *i* that is with individuals in group *j*. In this case, the expression in (1) can be written as

(7) $$\lambda (\alpha )=\lambda_{i}≐a_{i}\beta_{i}\overset{n}{\underset{j=1}{\sum }}c_{ij}\frac{I_{i}}{N_{j}},\;\text{for }\alpha_{i-1}\leq \alpha <\alpha_{i},\;i=1,2,\ldots ,n,$$

#### 2.2.1 No vaccination

Let *W*_*i*_ = *α*_*i*_ − *α*_*i*−1_ denote the width of age group *i*. Note that, for *α* = *α*_*i*_,

(8) $$e^{-\int_{0}^{\alpha }\lambda (s)ds}=e^{-\sum_{k=1}^{i}\int_{\alpha_{k-1}}^{\alpha_{k}}\lambda (s)ds}=e^{-\sum_{k=1}^{i}\lambda_{k}W_{k}}.$$

Let $S_{i}$ denote the proportion of sero-positive individuals in age group *i*, *i* = 1, 2, …, *n*. The probability of not having been infected up to age *α*_*i*_ is

$$e^{-\sum_{k=1}^{i}\lambda_{k}W_{k}},\;i=1,2,\ldots ,n.$$

From (8), the probability of being infected at age *α*_*i*_ is

(9) $$S_{i}=1-e^{-\sum_{k=1}^{i}\lambda_{k}W_{k}},\;i=1,2,\ldots ,n,$$

from which we have

$$\overset{i}{\underset{k=1}{\sum }}\lambda_{k}W_{k}=-\text{ln}\left(1-S_{i}\right).$$

It follows that $\lambda_{1}=-\left[\text{ln}\left(1-S_{1}\right)\right]/W_{1}$ and

(10) $$\begin{array}{l} \lambda_{i}=\frac{1}{W_{i}}\left[\overset{i}{\underset{k=1}{\sum }}\lambda_{k}W_{k}-\overset{i-1}{\underset{k=1}{\sum }}\lambda_{k}W_{k}\right],\\ =\frac{1}{W_{i}}\left[\text{ln}\left(\frac{1-S_{i-1}}{1-S_{i}}\right)\right],\;i=2,3,\ldots ,n. \end{array}$$

**Figure 2:**
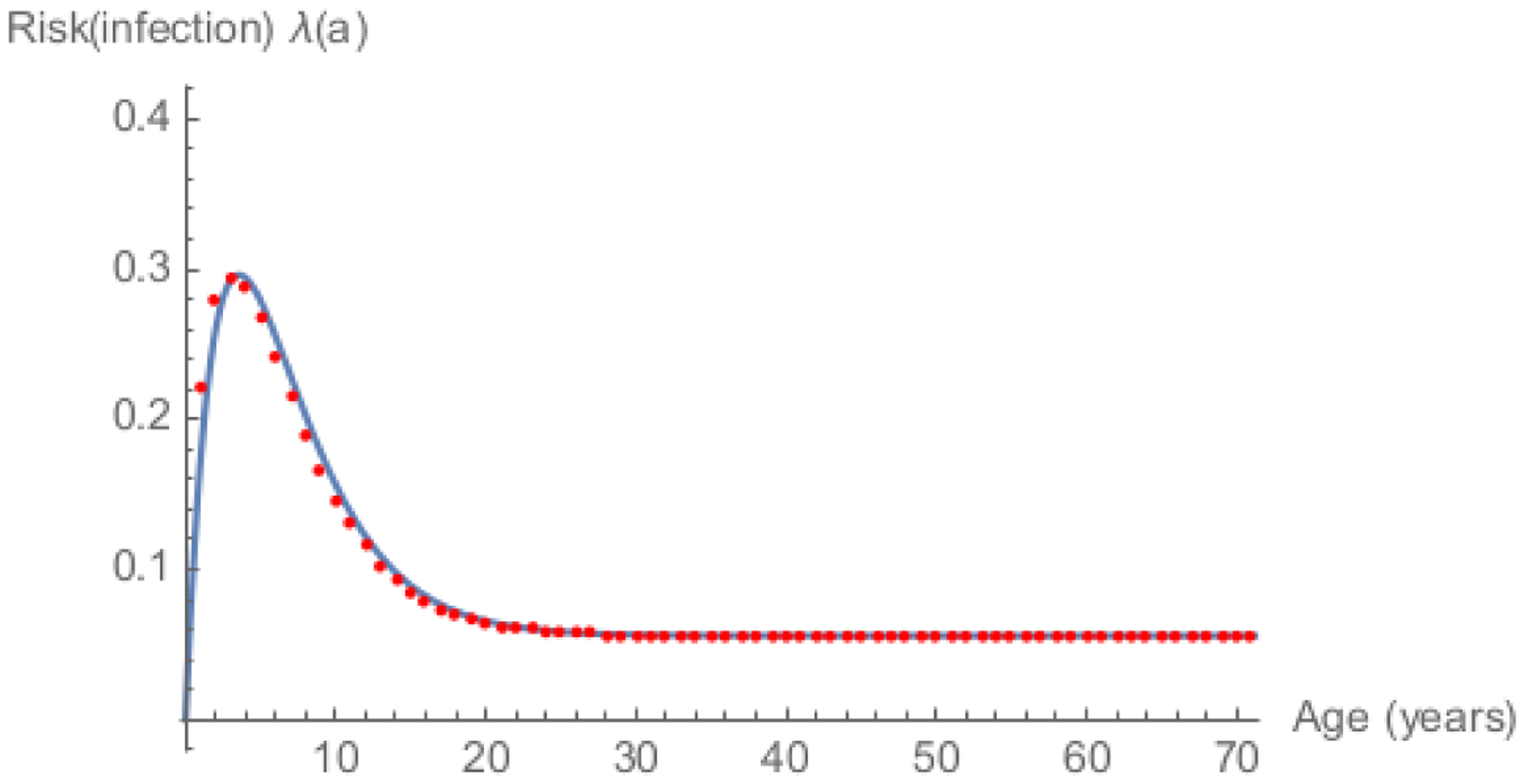
Comparison between the curve in Figure 1B and the *λ*_*i*_ values (the dots) calculated using (10) in which $S_{i}$ are generated from the function *F*(*α*) in Figure 1A.

Figure 2 compares the curve in Figure 1B and the *λ*_*i*_ values calculated using (10) in which the $S_{i}$ are generated from the function *F*(*α*) in Figure 1A.

#### 2.2.2 Vaccination at birth

Let *q*_*i*_ denote the proportion of individuals who are *not* immune due to vaccination at birth (group 1), *i* = 1, 2, …, *n*. Note that the probability of having been neither vaccinated nor infected before age *α*_*i*_ is

$$q_{i}e^{-\sum_{k=1}^{i}\lambda_{k}W_{k}},\;i=1,2,\ldots ,n.$$

Thus, the probability of being sero-positive at age *α*_*i*_ is

(11) $$S_{i}=1-q_{i}e^{-\sum_{k=1}^{i}\lambda_{k}W_{k}},\;i=1,2,\ldots ,n,$$

from which we have that

$$\overset{i}{\underset{k=1}{\sum }}\lambda_{k}W_{k}=\text{ln}\left(\frac{q_{i}}{1-S_{i}}\right),\;i=1,2,\ldots ,n.$$

Then,

$$\lambda_{1}=\frac{1}{W_{1}}\left[\text{ln}\left(\frac{q_{1}}{1-S_{1}}\right)\right],$$

and

(12) $$\begin{array}{l} \lambda_{i}=\frac{1}{W_{i}}\left[\sum_{k=1}^{i}\lambda_{k}W_{k}-\sum_{k=1}^{i-1}\lambda_{k}W_{k}\right],\\ =\frac{1}{W_{i}}\left[\text{ln}\left(\frac{q_{i}}{1-S_{i}}\right)-\text{ln}\left(\frac{q_{i-1}}{1-S_{i-1}}\right)\right],\\ =\frac{1}{W_{i}}\left[\text{ln}\left(\frac{1-S_{i-1}}{1-S_{i}}\cdot \frac{q_{i}}{q_{i-1}}\right)\right],\;i=2,3,\ldots ,n. \end{array}$$

#### 2.2.3 Supplementary immunization

Let *σ*_*i*_ denote the vaccination (immunization) rate of group *i* due to a supplementary immunization program, *i* = 1, 2, …, *n*. Then the probability of neither being vaccinated nor infected before age *α*_*i*_ is

$$q_{i}e^{-\sum_{k=1}^{i}\left(\lambda_{k}+\sigma_{k}\right)W_{k}},\;i=1,2,\ldots ,n,$$

and the probability of being sero-positive at age *α*_*i*_ is

(13) $$S_{i}=1-q_{i}e^{-\sum_{k=1}^{i}\left(\lambda_{k}+\sigma_{k}\right)W_{k}},\;i=1,2,\ldots ,n.$$

From equation (13), we have

$$\overset{i}{\underset{k=1}{\sum }}\left(\lambda_{k}+\sigma_{k}\right)W_{k}=\text{ln}\left(\frac{q_{i}}{1-S_{i}}\right),$$

from which we obtain

$$\lambda_{1}=\frac{1}{W_{1}}\left[\text{ln}\left(\frac{q_{1}}{1-S_{1}}\right)\right]-\sigma_{1},$$

and

(14) $$\begin{array}{l} \lambda_{i}=\frac{1}{W_{i}}\left[\overset{i}{\underset{k=1}{\sum }}\left(\lambda_{k}+\sigma_{k}\right)W_{k}-\overset{i-1}{\underset{k=1}{\sum }}\left(\lambda_{k}+\sigma_{k}\right)W_{k}\right]-\sigma_{i},\\ =\frac{1}{W_{i}}\left[\text{ln}\left(\frac{q_{i}}{1-S_{i}}\right)-\text{ln}\left(\frac{q_{i-1}}{1-S_{i-1}}\right)\right]-\sigma_{i},\\ =\frac{1}{W_{i}}\left[\text{ln}\left(\frac{1-S_{i-1}}{1-S_{i}}\cdot \frac{q_{i}}{q_{i-1}}\right)\right]-\sigma_{i},\;i=2,3,\ldots ,n. \end{array}$$

## 3 Estimating the FOI

### 3.1 Estimating the FOI from disease surveillance

**Figure 3:**
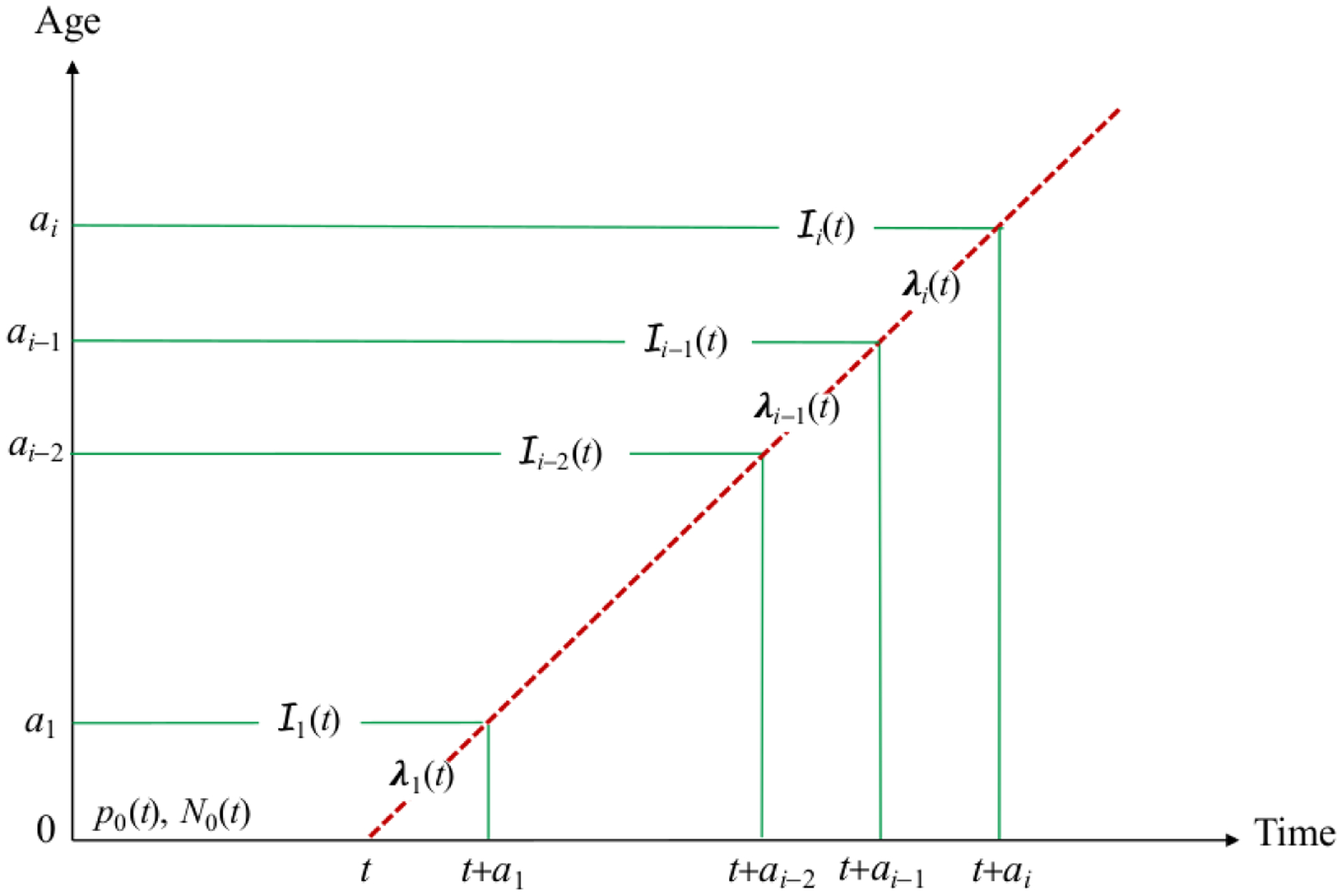
Depiction of group-specific surveillance $\left(I_{i}\right)$ and the FOI (*λ*_*i*_) for a cohort born (i.e., aged 0) at time *t* with proportion *p*_0_(*t*) of *N*_0_(*t*) immunized.

Consider a cohort born at time *t* of size *N*_0_(*t*) and immunization (uptake × efficacy) proportion *p*_0_(*t*). For this cohort, introduce the following notation:

$I_{i}(t)$ is the number of new infections in age group *i* from disease surveillance (adjusted for estimated under-reporting); i.e., people aged [*a*_*i*−1_, *a*_*i*_) who were infected during the period *t* + *a*_*i*−1_ to *t* + *a*_*i*_ (see Figure 3);
*λ*_*i*_(*t*) is the force or hazard rate of infection for people aged [*a*_*i*−1_, *a*_*i*_);
*p*_0_(*t*) is the proportion of the *N*_0_(*t*) people aged [0, *a*_1_) at time *t* that is immunized.

Then, within this cohort, the total number of infected people aged *a* < *a*_*i*−1_ at time t + *a*_*i*−1_ is $\sum_{k=1}^{i-1}I_{k}(t)$, and the number of susceptible people in age group *i* − 1 at time *t* + *a*_*i*−1_ is

$$N_{0}(t)\left[1-p_{0}(t)\right]-\overset{i-1}{\underset{k=1}{\sum }}I_{k}(t).$$

Let *W*_*i*_ = *a*_*i*_ − *a*_*i*−1_. Then,

$$\lambda_{i}(t)\left(N_{0}(t)\left[1-p_{0}(t)\right]-\overset{i-1}{\underset{k=1}{\sum }}I_{k}(t)\right)W_{i}=I_{i}(t),$$

from which we obtain the FOI for age group *i*:

(15) $$\lambda_{i}(t)=\frac{I_{i}(t)}{\left(N_{0}(t)\left[1-p_{0}(t)\right]-\sum_{k=1}^{i-1}I_{k}(t)\right)W_{i}},\;i=1,2,\ldots ,n.$$

**Figure 4:**
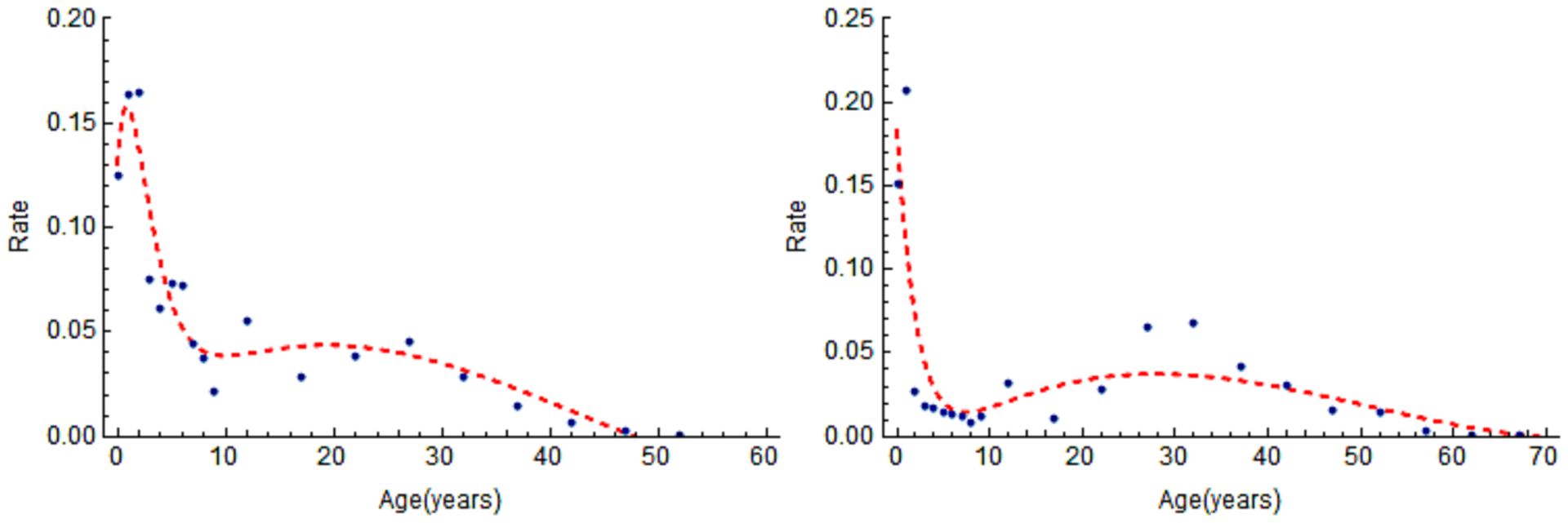
FOI among persons susceptible to measles by age in China during 2006 (A) and 2014 (B) estimated via equation (15). Source: [6].

Figure 4 shows results presented in [6] illustrating use of equation (15) to estimate the FOI *λ*_*i*_ based on measles surveillance data $\left(I_{i}\right)$ in China during 2006 and 2014.

### 3.2 Estimating the FOI from serology

**Figure 5:**
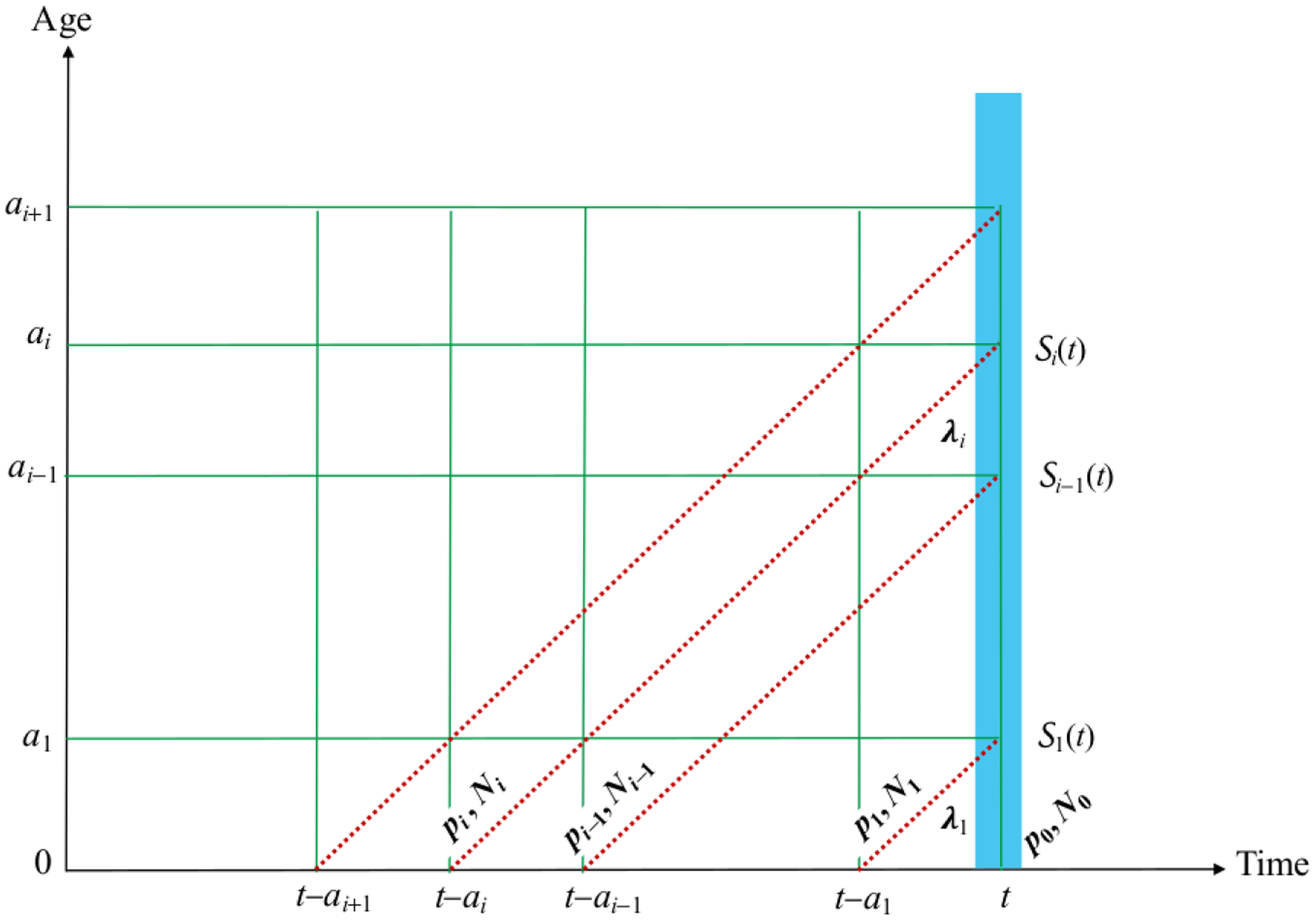
Depiction of the approach using group-specific cross-sectional serological observations at time *t*. $S_{i}(t)$ denotes the proportion of sero-positive people in age group *i* at time *t*, which includes those who were immunized at time *t* − *a*_*i*_ with proportion *p*_*i*_ of a population *N*_*i*_ and infected with FOI *λ*_*i*_ in $\left(1-S_{i-1}\right)N_{i-1}$.

Given proportions with antibodies from a cross-sectional serological survey at time *t* (i.e., $S_{i}(t)$ are available), the FOI may be estimated independent of disease surveillance. Note that we can write cumulative infections at ages *a*_*i*_ and *a*_*i*−1_ at time *t* as

$$\left[S_{i}-p_{i}\right]N_{i}\;\text{and}\;\left[S_{i-1}-p_{i-1}\right]N_{i-1},\;i>1,$$

where *p*_*i*_ is the proportion of people in age group *i* who were immunized at birth (i.e., at time *t*−*a*_*i*_), and *N*_*i*_ is the number of people in age groups *i* (see Figure 5). Then

$$\lambda_{1}=\frac{\left(S_{1}-p_{1}\right)N_{1}}{\left(1-p_{0}\right)N_{0}},$$

and

$$\lambda_{i}=\frac{\left[S_{i}-p_{i}\right]N_{i}-\left[S_{i-1}-p_{i-1}\right]N_{i-1}}{\left(1-S_{i-1}\right)N_{i-1}},\;i=2,3,\ldots ,n.$$

## 4 Estimating the FOI when immunity wanes

One difference between viral and bacterial pathogens is that infections with the former usually do and latter do not generate permanent immunity; and thus, multiple infections in a lifetime may be possible. The example in this section is from [3], who used antibody concentrations to pertussis toxin above 100 or 150 IU per ml in Sweden to estimate the FOI. The formulas for probabilities of having had one, two, and three infections by age *α* are derived in [3] and [8].

Let *F*(*α*) denote the cumulative probability of infection at age *α* and let *λ*(*α*) denote the hazard rate of infection at age *α*. If only one infection is possible in a lifetime, then

(16) $$F(\alpha )=q\left(1-e^{\int_{0}^{\alpha }\lambda (u)du}\right)+p\int_{0}^{\alpha }\omega (s)e^{-\omega s}\left(1-e^{\int_{s}^{\alpha }\lambda (u)du}\right)ds,$$

where *p* is the proportion of infants immune by virtue of passively acquired maternal antibodies, *q* = 1 − *p* is the proportion of infants susceptible at birth, and *ω*(*r*) is the rate of immunity waning at age *r*. When *p* = 0, equation (16) is the same as that given in [1].

In [1] is assumed that the FOI had the following functional form:

(17) $$\lambda (\alpha )=(a\alpha -c)e^{-ba}+d,$$

where *a*, *b*, *c* and *d* are constants. This assumption is useful where observations are few or highly variable.

**Figure 6:**
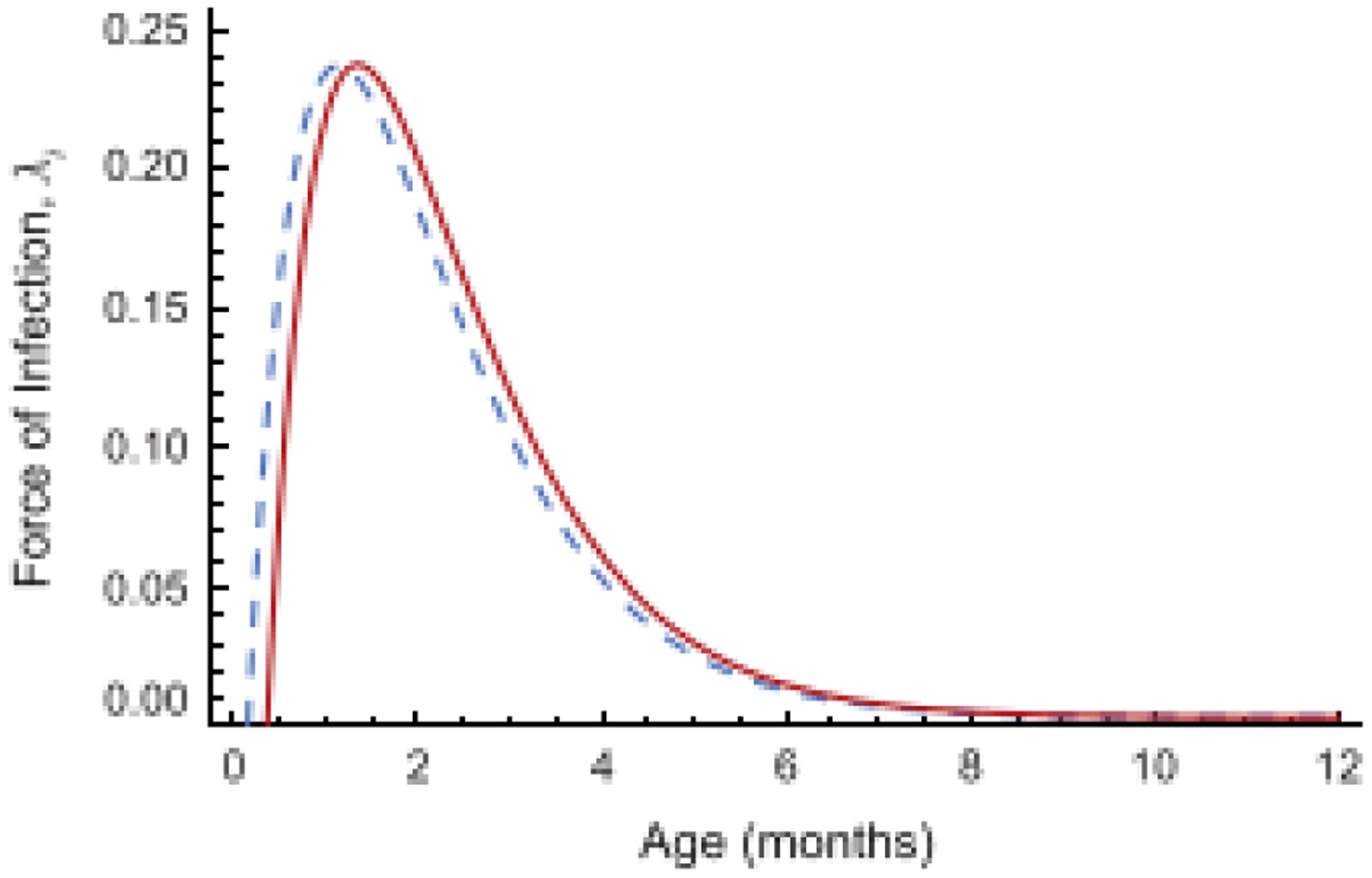
Fits of equation (16) with *q* = 1 (dashed curve) and *q* = 0.483 (solid curve) to age specific proportions of preschool children with anti-PT titers ≥ 10 EU/ml. Source: [3].

In [3], Feng, et al. fit equation (16) with *λ*(*α*) given by (17) to proportions of preschool children with antibodies to pertussis toxin greater than 10 IU per ml to estimate the constants in *λ*(*α*). The result is illustrated in Figure 6, which shows the FOI in two cases: (i) *q* = 1 and (ii) *q* = 0.483. The estimated parameter values for the FOI in (17) are (i) *a* = 0.712, *b* = 1, *c* = 0.082, *d* = 0.002) (the dashed curve in (b)), and (ii) for the case with maternal antibodies, *p* = 0.483, *a* = 0.884, *b* = 1, *c* = 0.291, *d* = 0.002 (the solid curve in (b)).

Suppose that people can be infected twice in a lifetime. Introduce the following notation:

$P_{S_{1}}(\alpha )$ is the probability of remaining susceptible from birth to at age *α*, given by $e^{-\int_{0}^{\alpha }\lambda (u)du}$;
$P_{S_{2}}(\tau )$ is the probability of remaining susceptible *τ* time units after recovering from the first infection;
$P_{I_{i}}(\tau )$ is the probability of remaining infected *τ* time units after the *i*th infection (*i* = 1, 2);
$P_{R_{i}}(\tau )$ is the probability of remaining immune *τ* time units after recovery from the *i*th infection (*i* = 1, 2). $P_{R_{2}}(\tau )=1$ if only two infections in a lifetime.

**Figure 7:**
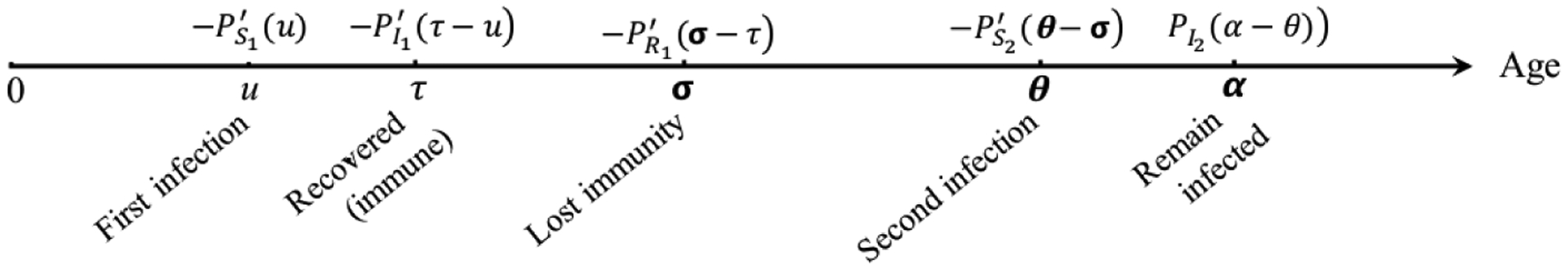
Diagram showing the order of events for 2 infections.

Assume that people were first infected at age *u*, recovered (and became immune) at age *τ* > *u*, lost immunity (and became susceptible again) at age *σ*, were re-infected at age *θ*, and remain infected at age *α* (see Figure 7). Then the cumulative probability of infection at age *α* is

(18) $$F(\alpha )=I_{1}(\alpha )+I_{2}(\alpha ),$$

where

(19) $$I_{1}(\alpha )=\int_{0}^{\alpha }\lambda (u)e^{-\int_{0}^{u}\lambda (r)dr}P_{I_{1}}(\alpha -u)du,$$

and

(20) $$I_{2}(\alpha )=\int_{0}^{\alpha }\int_{0}^{\theta }\int_{0}^{\sigma }\int_{0}^{\tau }\left[-P_{S_{1}}^{\prime }(u)\right]\left[-P_{I_{1}}^{\prime }(\tau -u)\right]\left[-P_{R_{1}}^{\prime }(\sigma -\tau )\right]\times \left[-P_{S_{2}}^{\prime }(\theta -\sigma )\right]P_{I_{2}}(\alpha -\theta )dud\tau d\sigma d\theta ,$$

represent probabilities of first and second infection at age *α*, respectively.

Consider the special case when the sojourns in *I*_*i*_ and *R*_1_ stages are exponentially distributed; i.e.,

$$P_{I_{i}}(\tau )=e^{-\gamma \tau },\;P_{R_{1}}(\tau )=e^{-\omega \tau },$$

where 1/*γ* and 1/*ω* are mean periods of infection and immunity. Assume that the FOI for the second infection is *ρλ*(*α*) where 0 < *ρ* < 1 indicates a possible diminution in the rate of re-infection. Then the expression for *I*_2_(*a*) in (20) becomes

(21) $$I_{2}(\alpha )=\int_{0}^{\alpha }\int_{0}^{\theta }\int_{0}^{\sigma }\int_{0}^{\tau }\lambda (u)e^{-\int_{0}^{u}\lambda (r)dr}\gamma e^{-\gamma (\tau -u)}\omega e^{-\omega (\sigma -\tau )}\times \rho \lambda (\theta )e^{-\int_{\sigma }^{\theta }\rho \lambda (r)dr}e^{-\gamma (\alpha -\theta )}dud\tau d\sigma d\theta .$$

**Figure 8:**
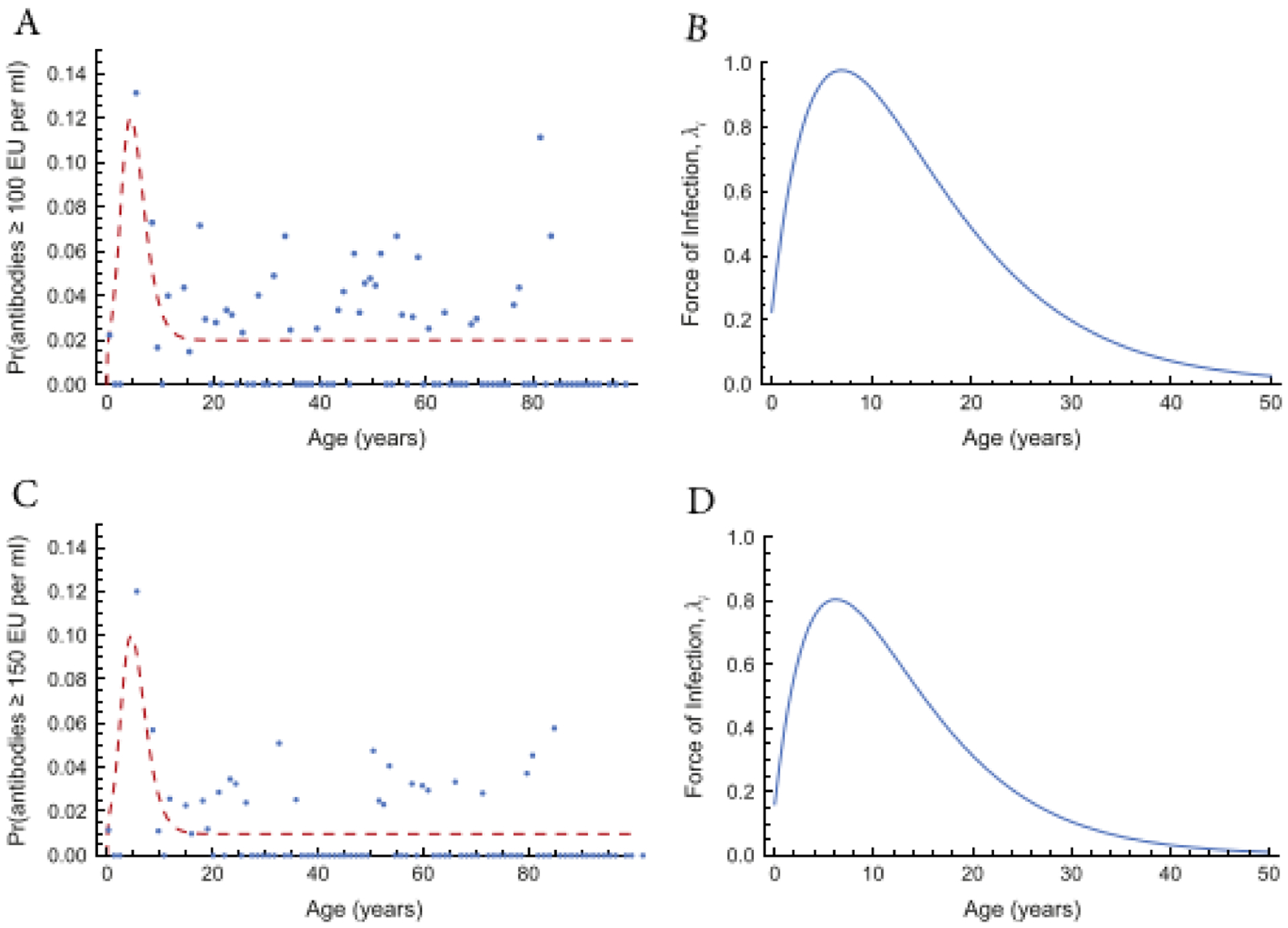
Age-specific proportions of sera containing antibodies to pertussis toxin above 100 EU/ml (A and B) and above 150 EU/ml (C and D) and fitted equations (19) and (21). The parameters for the corresponding FOI *λ*_*i*_ are (B) *a* = 0.314, *b* = 0.13, *c* = −0.225, *d* = 0.001, and (D) *a* = 0.301, *b* = 0.149, *c* = −0.16, *d* = 0.001. Source: [3].

Figure 8 shows the result of fitting equations (19) and (21) to age-specific proportions of persons whose sera contain antibodies to pertussis toxin above 100 EU/ml (A and B) and 150 EU/ml (C and D). The estimated parameter values for the FOI are *a* = 0.314, *b* = 0.13, *c* = −0.225, *d* = 0.001 (in B) and *a* = 0.301, *b* = 0.149, *c* = −0.16, *d* = 0.001 (in D).

In [8] Wang, et al. considered the case of three infectious in a lifetime. Let *z*_1_(*α*) denote the probability that a person born susceptible is first infected at age *α*, *z*_2_(*α*) denote the probability that a person aged *α* either was born susceptible and infected a second time, or born with maternal antibodies and first infected after losing maternal immunity, *z*_3_(*α*) denote the probability that a person aged *α* either was born with maternal antibodies and had a second infection or was born susceptible and had a third infection. Then the cumulative probability of infection at age *α* is given by

$$F(\alpha )=z_{1}(\alpha )+z_{2}(\alpha )+z_{3}(\alpha ),$$

where

$$\begin{array}{l} z_{1}(\alpha )=q\int_{0}^{\alpha }\lambda (u)e^{-\int_{0}^{u}\lambda (r)dr}e^{-\gamma (\alpha -u)}du,\\ z_{2}(\alpha )=p\int_{0}^{\alpha }\int_{0}^{\theta }\left[\omega e^{-\omega u}\right]\left[\rho \lambda (\theta )e^{-\int_{u}^{\theta }\rho \lambda (r)dr}\right]\left[e^{-\gamma (\alpha -\theta )}\right]\;dud\theta \\ +q\int_{0}^{\alpha }\int_{0}^{\theta }\int_{0}^{\sigma }\int_{0}^{\tau }\lambda (u)e^{-\int_{0}^{u}\lambda (r)dr}\gamma e^{-\gamma (\tau -u)}\omega e^{-\omega (\sigma -\tau )}\\ \times \rho \lambda (\theta )e^{-\int_{\sigma }^{\theta }\rho \lambda (r)dr}e^{-\gamma (\alpha -\theta )}dud\tau d\sigma d\theta , \end{array}$$

and

$$\begin{array}{l} z_{3}(\alpha )=p\int_{0}^{\alpha }\int_{0}^{\theta }\int_{0}^{\sigma }\int_{0}^{\tau }\int_{0}^{\zeta }\left[\omega e^{-\omega u}\right]\left[\rho \lambda (\zeta )e^{-\int_{u}^{\zeta }\rho \lambda (r)dr}\right]\left[\gamma e^{-\gamma (\tau -\zeta )}\right]\\ \times \left[\omega e^{-\omega (\sigma -\tau )}\right]\left[\rho \lambda (\theta )e^{-\int_{\sigma }^{\theta }\rho \lambda (r)dr}\right]\left[e^{-\gamma (\alpha -\theta )}\right]dud\zeta d\tau d\sigma d\theta \\ +q\int_{0}^{\alpha }\int_{0}^{\theta }\int_{0}^{\sigma }\int_{0}^{\tau }\int_{0}^{\zeta }\int_{0}^{\psi }\int_{0}^{\chi }\left[\lambda (u)e^{-\int_{0}^{u}\lambda (r)dr}\right]\left[\gamma e^{-\gamma (\chi -u)}\right]\left[\omega e^{-\omega (\psi -\chi )}\right]\\ \times \left[\rho \lambda (\zeta )e^{-\int_{\psi }^{\zeta }\rho \lambda (r)dr}\right]\left[\gamma e^{-\gamma (\tau -\zeta )}\right]\left[\omega e^{-\omega (\sigma -\tau )}\right]\left[\rho \lambda (\theta )e^{-\int_{\sigma }^{\theta }\rho \lambda (r)dr}\right]\\ \times \left[e^{-\gamma (\alpha -\theta )}\right]dud\chi d\psi d\zeta d\tau d\sigma d\theta . \end{array}$$

**Figure 9:**
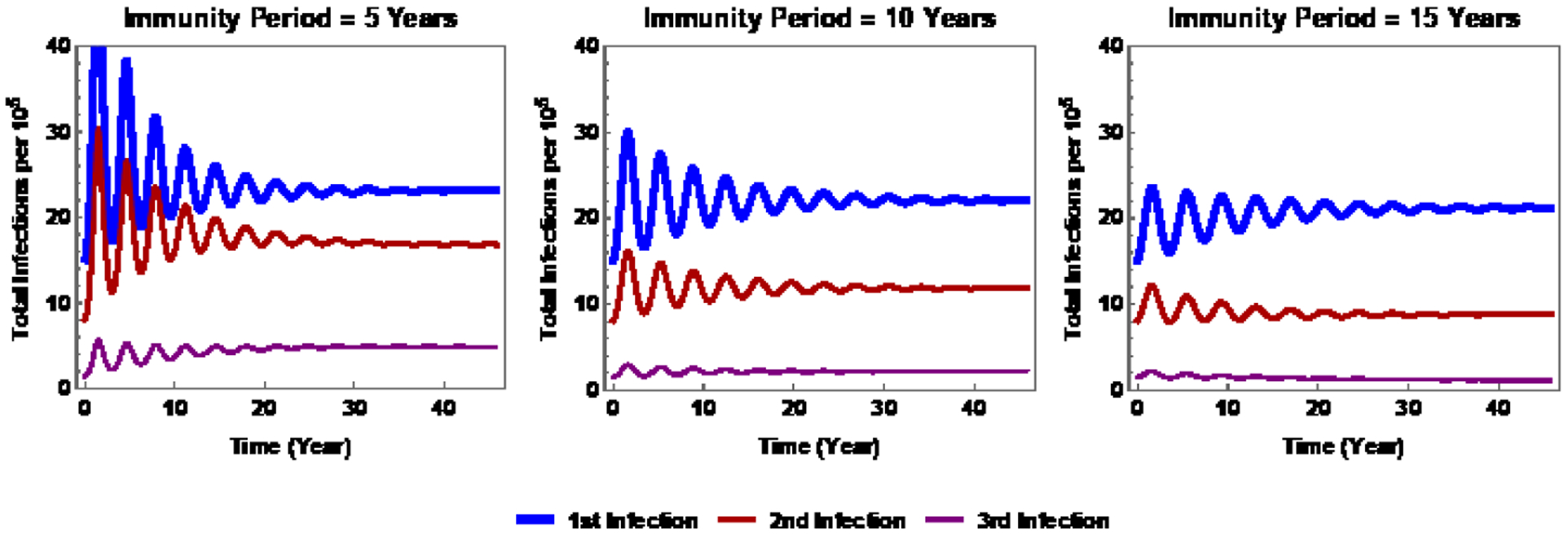
Numerical simulations of the age-dependent ODE system for three values of the immunity period: 1/*ω* = 5, 10, and 15 years. Source: [8].

The numerical simulations of [8] suggest that a model with two infections suffices (although some questions may require more than two). Figure 9 is based on the reduction of the 3-infection PDE model to an ODE model with aging by assuming piecewise constant parameter functions (see [8] for more details). This figure shows immunity periods of 5, 10, and 15 years. We observe that the proportions of people with three infections in a lifetime is much lower than those with one or two infections, particularly when immunity is long-lasting.

## 5 Discussion

In this paper, we derive formulas that can be used to estimate age-dependent hazard rates of infection or FOI by fitting to observed serology or disease surveillance. Expressions for the FOI for continuous *λ*(*a*) and discrete age *λ*_*i*_ are presented. And several examples are shown of fitting these formulas to observations of varicella, measles, and pertussis. These FOI are needed to estimate the probability of infection on contact *β*_*i*_ using relations like equation (2). We have not included measures of uncertainty associated with our best fitting parameter estimates, as this subject warrants separate treatment.

The cases considered in this paper include those when routine and/or supplementary vaccination programs are implemented, and diseases confer permanent or temporary immunity. Although we consider relatively simple scenarios; e.g., at most three infections in a lifetime for pertussis, our approach can be used to derive formulas for the FOI if more than three infections are considered. However, numerical simulation results shown in Figure 9 suggest that it may be sufficient to consider only two infections.
